# Psychotropic Medications Use among Children with Autism in Saudi Arabia

**DOI:** 10.3390/children9070966

**Published:** 2022-06-28

**Authors:** Shuliweeh Alenezi, Fahad Alnemary, Asma Alamri, Dalal Albakr, Lamees Abualkhair, Faisal Alnemary

**Affiliations:** 1 Department of Psychiatry, College of Medicine, King Saud University, Riyadh 11451, Saudi Arabia; lamees.f.a@gmail.com; 2 Department of Special Education, College of Education, Taif University, Taif 21944, Saudi Arabia; fanemary@tu.edu.sa; 3 Department of Child Psychiatry, King Abdullah Specialist Children Hospital, Riyadh 14611, Saudi Arabia; asmaa.s.alamri@gmail.com; 4 Department of Psychiatry, Imam Abdulrahman Bin Faisal University, Dammam 34212, Saudi Arabia; albakrdm@gmail.com; 5 Autism Center of Excellence, Riyadh 12713, Saudi Arabia; falnemary@acesaudi.org

**Keywords:** autism spectrum disorder, psychotropic medications, Saudi Arabia

## Abstract

Psychotropic medication use is rising among children with autism spectrum disorders (ASD) in Saudi Arabia. Two hundred ninety-three parents of children diagnosed with ASD completed a parental concerns questionnaire (PCQ) online to examine children’s family socio-demographics, health conditions and comorbidities, and past and current exposure to psychotropic medication as prescribed by their primary doctor. Findings revealed that more than one-third of the parents (39.08%) reported that their children were using medications at the time of the survey; risperidone (53%), methylphenidate (30%), and valproic acid (9%) were the most commonly used. A smaller number of parents stated that their children had previously used medications (16.09%). The most often prescribed drugs among this group were risperidone (45%), followed by methylphenidate (32%) and valproic acid (17%). The variables that showed a statistically significant association with the current use of psychotropic drugs were the child’s age (OR = 1.25, 95% CI: 1.12, 1.40, *p* < 0.001), presence of comorbidities (OR = 7.75, 95% CI: 3.48, 17.24, *p* < 0.001), communication difficulties (OR = 1.79, 95% CI: 1.09, 2.95, *p* < 0.021), and anxiety symptoms (OR = 1.70, 95% CI: 1.00, 2.87, *p* < 0.049). Similarly, the child’s age (OR = 1.23, 95% CI: 1.08, 1.40, *p* < 0.002) and presence of comorbidities (OR = 2.83, 95% CI: 1.16, 6.29, *p* < 0.022) showed statistically significant associations with previous use.

## 1. Introduction

Increased awareness about autism spectrum disorder (ASD) in Saudi Arabia over the last two decades has improved services and interventions offered for this lifelong neurodevelopmental disorder, including psychotropic medication. Evidence suggests an early implementation of behavioral therapies might help alleviate core ASD symptoms and improve the trajectory of ASD’s impact on functional outcomes [1]. Furthermore, evidence indicates that the most beneficial approach is when behavioral therapies are combined with careful psychopharmacological monitoring [2]. However, only two medicines, risperidone and aripiprazole, have been approved for noncore ASD symptoms (irritability) in ASD [3,4]. Despite this, a systematic analysis found widespread prescription medication usage in children with ASD, with a median frequency of 41.9% in children with increasing prescription medication use linked to psychiatric comorbidities [5]. The most commonly prescribed medications are atypical antipsychotics followed by stimulant and nonstimulant attention deficit/hyperactivity disorder medications and antidepressants [6]. While psychotropic medications are an effective part of the treatment for individuals with ASD, challenges continue over their side effects and need for monitoring [7].

Taking a closer look, in Saudi Arabia, the prevalence of ASD is one per 167, suggesting that the total number of individuals with ASD is over 167,000 [8]. Data about the confirmed cases of ASD in Saudi Arabia are not available, and anecdotal data suggest that many children with ASD have not been identified yet. Only one recent report on a hospital-based sample indicated a prevalence of 2.5% [9]. While ASD services are continually improving in Saudi Arabia, existing reports indicate that some parents pay and/or travel to access services for their children with ASD. Newer reports show that children use a wide range of interventions, including psychopharmacological interventions [10]. However, research is still growing in this part of the world and providing systematic information about the management of ASD with no specific studies investigating the use of psychotropic medication or associated predictors among Saudi children.

However, the prevalence and patterns of medication use among individuals with ASD have been investigated in different countries. For example, in South Africa, in a survey-based study of children and adolescents with ASD, parents indicated that 24.6% of the 65 children used psychotropic medications, and antipsychotics were the most commonly used psychotropics, followed by stimulants, antidepressants, and mood stabilizers [11]. In Iran, 345 families were interviewed about the use of psychotropic medication among their children with ASD [12]. This study revealed that 80% of participants were using at least one psychotropic medication at the study time. In comparison, up to 97% of the sample had at least one psychotropic medication, and the most frequently used medications were antipsychotics (57.4%), followed by antidepressants (8.7%). Another study from Italy examined the prevalence and predictors of psychotropic medication use in 195 children with ASD aged 14–58 and showed that more than half of the children used at least one medication; antipsychotics were prescribed to 40%, and 30% of the sample were on anticonvulsants/mood stabilizers [13]. However, epilepsy, psychiatric comorbidities, and the severity of repetitive behaviors predicted psychotropic medication use in this study. Similarly, two studies in Ohio and North Carolina surveyed families to assess the prevalence and patterns of psychotropic medication use among children with autism [14,15]. Results showed that nearly half of children were taking some form of psychotropic agent, and the most common psychotropic agents included antidepressants, antipsychotics, antihypertensives, and stimulants. In addition, greater age, more severe autism, more severe intellectual handicap, and housing outside the family home were associated with medication use.

As no studies have been conducted in Saudi Arabia, there is a need to examine the prevalence and patterns of medication use among individuals with ASD to inform the rapidly growing ASD field. Leveraging an existing dataset, we aimed to describe the current and previous use of psychotropics among children with ASD in Saudi Arabia and associated sociodemographic and comorbidity variables.

## 2. Methods

### 2.1. Data Source and Sampling 

Due to the lack of accurate statistics for children with ASD in Saudi Arabia, a convenience sample was drawn for this study. Survey data were collected as part of an effort to examine the use and quality of services for children diagnosed with ASD, using an online platform (Qualtrics ©) from 22 February to 31 May 2017. Participants were recruited via email sent by the Department of Special Education in the Ministry of Education to all school districts in the country (*n* = 46). The message included a brief description of the survey, along with its URL and a request to share the survey’s link with schools that serve students with ASD in that district. The same message was shared with supervisors of schools serving students with ASD. Another link to the survey was made available on the Twitter account of the Center for Autism Research, King Faisal Specialist Hospital & Research Center in Riyadh. Participants were asked to complete the survey for their oldest child with ASD.

### 2.2. Survey Description

The survey was developed based on previous studies and anecdotal reports from clinical experts and families in Saudi Arabia. Apart from PCQ, all other parts of the survey were originally developed in English and then translated into Arabic using the forward-translation method by Hambelton & Kanjee, 1995, to improve the cross-cultural validity. An experienced bilingual committee (*n* = 4; Saudi graduate students in the field of Special Education who were fluent in both languages) translated the survey into Arabic. The committee members worked individually to translate the survey, and then they met to discuss their translations and created one Arabic version. The purpose of this process was to make the Arabic version of the survey meaningful rather than develop items that translate exactly as in the English version. The survey included the following sections:

#### 2.2.1. Family Characteristics

This section served to gather information about the family, including the parents’ level of education and annual household income. Income was coded based on the sufficiency line in Saudi Arabia [16], which is considered the income a family requires to meet its needs without public support. These needs include housing, childcare, food, health care, transportation, and entertainment. We collapsed the variable into three categories: below the sufficiency line, from the sufficiency line to 100% above, and >100% above the sufficiency line.

#### 2.2.2. Child Characteristics

This part of the survey asked parents to provide information about their child, including age and gender, comorbidity, and severity of symptoms. Comorbidity was measured with a list for variables, which appeared to parents if they answered yes to “intellectual disability”, “attention deficit/hyperactivity disorder (ADHD)”, “depression”, “epilepsy”, or “other”.

#### 2.2.3. Parents Report of Severity of Symptoms

This section is a composite variable that combines parent responses to the parental concerns questionnaire (PCQ), a measure that assesses the extent to which each of several core and behavioral symptoms of ASD have been a problem for their child [17]. The PCQ consists of 13 items (e.g., “My child does not use words or has difficulty initiating conversations”, “My child completes routines always in the same manner”, and “My child does not fall asleep easily and wakes often”) with response options of “strongly disagree”, “disagree”, “agree”, and “strongly agree”. The PCQ has adequate psychometric properties, and its factor structure has been examined previously in children with ASD in Saudi Arabia [18].

#### 2.2.4. Use of Medication

In this section, the parents answered two questions about medication use using free text. These questions asked what medications their child had used in the past and what medication they were using at the time of the survey.

## 3. Analysis

Descriptive information was calculated for the entire sample (numbers and percentages for count data and means and standard deviations for continuous variables). Logistic regressions on the use of medication (past and current) were conducted, adjusting for child and family characteristics. IBM SPSS 26 software for Windows was used for the analysis, and a *p*-value of <0.05 was considered significant.

## 4. Results

### 4.1. Submissions

A total of 375 surveys was completed during the four-month data collection period. A total of 133 surveys was accessed through the link that was shared with the Ministry of Education, and 242 surveys were completed through the link that was shared on the Twitter account of the Center for Autism Research. In total, 82 surveys contained missing data and were excluded from the sample. Therefore, the final sample included 293 surveys.

### 4.2. Sample Characteristics

Table 1 provides descriptive information of the sample. Most respondents were between 31 and 40 years old at the time of the survey, and 56% were mothers. Fairly higher maternal educational attainment was observed in this study, with 57% holding a four-year college degree or postgraduate degree. Thirty-six percent of the families had an annual household income below the sufficiency line. The median age of children was 7.5 years and ranged from 3 to 18 years (30% < 6 years; 47% aged 6–9; 13% 10–13 years; 10% 14–18 years).

### 4.3. Use of Medications

Table 2 shows that more than a third of the parents (39.08%) reported that their children were using medications at the time of the survey. The most commonly used medication was risperidone at 53%, followed by methylphenidate (30%) and valproic acid (9%). In addition, there was a smaller group indicating that their children had used medications in the past (16.09%). The most commonly used medication in this group was risperidone 45%, followed by methylphenidate 32% and valproic acid 17%.

### 4.4. Factors Associated with the Current Use of Medications

Multiple logistic regression was used to examine the association among the current use of psychotropic medications, sociodemographic factors, and PCQ scores (Table 3). The variables that showed a statistically significant association with current medication use were the child’s age, presence of comorbidity communication difficulties, and anxiety symptoms. For each year the child aged, the odds ratio of using psychotropic medications increased by 1.25 (OR = 1.25, 95% CI: 1.12, 1.40). Those with comorbidities had higher odds of currently using psychotropic agents (OR = 7.75, 95% CI: 3.48, 17.24) than those with no comorbidities. For children with communication difficulties, the odds of taking medication increased (OR = 1.79, 95% CI: 1.09, 2.95), and the same was observed for the presence of anxiety symptom (OR = 1.70, 95% CI: 1.00, 2.87).

### 4.5. Factors Associated with Past Use of Medications

Logistic regression of previous use of psychotropic medications showed a similar result as current use, i.e., child’s age and presence of comorbidities, and showed statistically significant relationships with previous use. For each year the child aged, the OR of previously using psychotropic medications increased by 1.23 (OR = 1.23, 95% CI: 1.08, 1.40). Those with comorbidities had higher odds of previously using psychotropic agents (OR = 2.83, 95% CI: 1.16, 6.29) than those with no comorbidities (Table 4).

## 5. Discussion

This study aimed to examine the use of medications and associated factors among children with ASD in Saudi Arabia. Surveying 293 parents who have experienced ASD services for their children (typically within the past six years) provides valuable insight into an essential group of families affected by ASD in Saudi Arabia. Findings from our study are somewhat consistent with previous research.

Our study found that psychotropic medications were used to manage noncore symptoms of ASD in 39.08% of children at the time of the survey and up to 16.09% of past use; this leaves only 39.9% of our sample that has never used psychopharmacological interventions. This finding, although surprising, mirrored what has been discovered in the literature that psychotropic medication use ranges from 2.7 percent to 80 percent. This suggests that pharmacological interventions can effectively manage noncore symptoms of ASD [5,12]. We believe this is probably related to the underdeveloped nonpharmacological services. However, current reports show that indications and determinant conditions for using psychotropic medications in children diagnosed with ASD vary among the children’s demographics and characteristics [19].

Research suggests that comorbidities are common in ASD; according to one study, 70% of people with ASD have at least one comorbid psychiatric condition [20]. Having comorbidity increases the likelihood of using psychotropic medications [21]. Parents reported comorbidity to be prevalent at 59.7% in our study. Furthermore, we found that comorbidities were associated more with current than past use (OR = 7.75 vs. OR = 2.83) of psychotropic medications. This finding was expected and confirmed by the results of other studies [22,23]. However, comorbidity with ADHD has been reported to be as high as 65 %; although it was only 45.4 % in our sample. Our study found a 16 % prevalence of intellectual disability, which is predicted to co-occur in approximately 26% of individuals with ASD [24]. Epilepsy was reported among 5% of our sample. Although this is lower than the literature’s reported range of 5–38 percent [25], it was close to the 9.3 percent found in a cross-sectional study in Saudi Arabia.

The literature shows that 80% of children with ASD experience clinically significant anxiety that worsens social deficits, daily living skills, and relationships with others [26]. Our findings were similar, with a significant association between current psychotropic medication use and a child being anxious in new or crowded places (OR = 1.70, 95% CI: 1.00, 2.87, *p* < 0.049). Although antidepressant medications used to treat comorbid ASD and anxiety are common, data supporting them are limited [26].

Interestingly, in our sample, having communication difficulties was significantly associated with the current use of psychotropics (OR = 1.79, 95% CI: 1.09, 2.95, *p* < 0.021). Although this association is not yet established, we believe this could be partially explained by the documented association of speech difficulties with externalizing and internalizing problems in kids with ASD [27].

Previous studies indicated that psychotropic medications were used to manage aggression, irritability, anxiety, and other behavioral disturbances [5]. Aggression was associated with the use of medications either now or in the past in our sample. However, aggression continues to be reported as one of the most common indications for starting antipsychotics [28]. A literature review revealed that 56% of children with ASD had directed aggression toward caregivers and 32% toward noncaregivers [29].

Children with ASD use medications nine times more often than the general population, and risperidone continues to be among the most commonly used [30]. In a recent study aiming to assess psychotropic prescription trends, risperidone was among the most highly used, accounting for 35% of prescriptions [31]. In our sample, current and past use of risperidone were 53% and 45%, respectively. This is higher than what was reported in the literature [32]. We believe that limited resources for behavioral interventions could explain this finding, with a limited number of centers and specialists offering nonpharmacological interventions. Risperidone is considered to effectively reduce behavioral problems, such as irritability, aggression, self-injurious behavior, and tantrums in children and adolescents with ASD, and is reported to have a good response in both short- and long-term outcomes and a tolerable side-effect profile [33]. The second most common class of medications prescribed in our sample was stimulants, with the most prevalent being methylphenidate, 30% for current use and 32% for previous use. Our findings were in a similar range to other studies [34]. This high use might indicate the high comorbidity of ASD and ADHD, which have been reported to be between 25.7% and 65% [35,36]. The least commonly used medication was valproic acid, a mood stabilizer, with 9% for current use and 17% for previous use. This result is lower than figures reported in a study showing that 29.2% of their sample took anticonvulsants/mood stabilizers; valproic acid was prescribed for 17% of their sample [5].

In terms of gender prevalence differences, our study showed that the ratio of affected males to females was four to one, consistent with epidemiological findings in Asia [37]. Furthermore, age and gender have been linked to an increased prevalence in psychotropic medication use, which typically was in older males. A systematic review of 15 studies showed that, as the child ages, there is a greater overall prevalence of psychotropic medication use [5]. The same review found that males were more commonly prescribed medication compared to females. The prevalence of males prescribed medication ranged from 75% to 92%, which resembles the percentage reported in our study at 80.5%. We also found that age was positively correlated with the past and more strongly with the current use of psychotropic interventions (*p*< 0.002 vs. *p* < 0.001). One explanation is that when a child becomes older, parents show less ability to control their behavior, especially if coupled with the limited accessibility of early intervention programs and behavioral therapies [38]. However, our logistic regression model could not confirm gender association due to the small sample size.

## 6. Limitations

This is the first study to examine medication use among children diagnosed with ASD in Saudi Arabia. Numerous limitations are noteworthy: First, this study is a secondary data analysis with a small sample size. Second, there is a lack of available similar studies in Saudi Arabia with which to compare our results. Third, the cross-sectional design limits the ability to generalize our findings on families of children with ASD. Fourth, findings in this study were based on parents’ reports, and as such, inaccurate reporting might have biased our findings. Finally, there is a lack of data on the indications of use of psychotropic medications, monitoring data, and responses from prescribing doctors’ perspectives.

Despite these limitations, this study provides a valuable picture of an important group of families: those affected by ASD in Saudi Arabia. Families of lower material educational attainments may have a lower-than-average knowledge about ASD and sophistication about ASD services. Additionally, children in this study had moderate to severe symptoms. Families of children with mild symptoms may have different experiences accessing and using ASD services.

## 7. Future Implications

Further research is needed that includes a nationally representative sample to confirm these findings and clarify comorbidities and to indicate the use of psychotropic medication in children who have ASD, including response to treatment and side effects.

## 8. Conclusions

The age of the child, the presence of comorbidities, communication challenges, and anxiety symptoms were the variables that indicated a significant association with current psychotropic drug use. These findings provide a valuable picture of families affected by ASD in Saudi Arabia. Additional research is warranted to better understand the factors that influence the use of medication in this population of children.

## Figures and Tables

**Table 1:** Demographic, health condition, and comorbidities of children based on parents’ report.

Characteristics	*n* (%)
Child	
Age of the child, mean	8.1 (4%)
Sex: male %	236 (80.5%)
Parental Concerns Questionnaire (PCQ) Items	
Anxiety symptom (Gets anxious in new places)	111 (42.7%)
Sleep disturbance (My baby is not sleeping easily or sleeping intermittently)	137 (52.6%)
Aggression (My child hits or bites others)	195 (74.8%)
Hyperactivity (My child is moving or running or continuously jumps)	74 (28.4%)
Inattentiveness (My child is having a hard time completing the tasks assigned to him)	62 (23.9%)
Irritability (My child changes moods / emotions)	73 (28.0%)
Communication issue (My child does not use words or finds it difficult to initiate talking to others)	58 (22.2%)
Inflexibility, routine maintenance (My child always completes routines in the same way)	93 (35.5%)
Sensitivity (My child is sensitive to light and sounds)	109 (41.7%)
Feeding problem (My child only eats certain types of food)	88 (33.7%)
Social difficulties (My child prefers to be alone or have few friends)	73 (28.0%)
Repetitive movement (My toddler is shaking or flapping their hands)	113 (43.4%)
Comorbidities (descriptive analysis)	175 (59.7)
No comorbidities	118 (40.3%)
ADHD	133 (45.4%)
Intellectual disability	47 (16%)
Epilepsy	17 (5.8%)
Others	26 (9.9%)
Paternal Educational Attainment	
<High school	32 (10.9%)
High school	58 (19.8%)
Some college credits	41 (14%)
College degree	126 (43%)
≥Graduate degree	36 (12.3%)
Maternal educational attainment	
<High school	36 (12.3%)
High school	72 (24.6%)
Some college credits	20 (6.8%)
College degree	144 (49.1%)
≥Graduate degree	21 (7.2%)
Annual household income	
Below sufficiency line	95 (32.2%)
From sufficiency line to 100% above	124 (42.3%)
>100% above sufficiency line	74 (25.3%)

**Table 2:** Comparison between current and past use of medication among participants.

# of Use of Psychotropic Medication *	Current Use		Past Use	
Medication	*n*	(%)	*n*	(%)
Risperidone	54	(53)	19	(45)
Methylphenidate	31	(30)	13	(32)
Valproic Acid	9	(9)	8	(17)
Carbamazepine	3	(3)	2	(4)
Atomoxetine HCI	2	(2)	1	(2)
Citalopram	2	(2)	1	(2)
Others	1	(1)	0	(0)
Total	102 (39.08%)		42 (16.09%)	

*, # of nonuse of psychotropic medication was 117 (39.9%).

**Table 3:** Multiple logistic regression for factors associated with the current use of psychotropic drugs.

Items	OR	*p*-Value	95% CI for OR	
			Lower	Upper
Child age	1.25	<0.001	1.12	1.40
Sex, male	1.19	0.706	0.49	2.87
Presence of comorbidity	7.75	<0.001	3.48	17.24
Paternal educational attainment				
<High school	REF			
High school	0.31	0.093	0.08	1.21
Some college credits	0.46	0.318	0.10	2.13
College degree	0.51	0.323	0.14	1.93
≥Graduate degree	0.44	0.310	0.09	2.16
Maternal educational attainment				
<High school	REF			
High school	1.25	0.717	0.37	4.17
Some college credits	0.24	0.203	0.03	2.17
College degree	1.19	0.773	0.36	3.91
≥Graduate degree	0.73	0.738	0.12	4.59
Annual household income				
Below sufficiency line	REF			
From sufficiency line to 100% above	1.70	0.234	0.71	4.05
>100% above sufficiency line	1.27	0.672	0.42	3.78
Parental Concerns Questionnaire (PCQ) Items				
My child does not use words or finds it difficult to initiate conversations with others.	1.79	0.021	1.09	2.95
My child always completes routines the same way.	0.93	0.775	0.57	1.52
My child gets anxious in new or crowded places.	1.70	0.049	1.00	2.87
My child is sensitive to light, sound, and touch.	0.66	0.101	0.40	1.08
My child does not sleep easily or their sleep is interrupted.	1.07	0.740	0.71	1.62
My child hits or bites others.	0.97	0.908	0.62	1.54
My child is constantly moving, running, or jumping.	1.02	0.923	0.63	1.67
My child has difficulty completing the tasks assigned to them.	1.14	0.621	0.68	1.91
My child has sudden changed moods/emotions.	1.07	0.802	0.63	1.81
My child only eats certain types of food.	0.88	0.542	0.57	1.34
My child prefers to be alone or have few friends.	0.80	0.416	0.48	1.36
My toddler is shaking or flapping their hands.	1.49	0.057	0.99	2.23

**Table 4:** Multiple logistic regression for the factors associated with previous use of psychotropic drugs.

Items	OR	*p*-Value	95% CI for OR	
			Lower	Upper
Child age	1.23	0.002	1.08	1.40
Sex, male	2.74	0.111	0.79	9.46
Severity level				
Mild	REF			
Moderate	1.28	0.804	0.18	9.00
Severe	0.76	0.794	0.10	5.93
Presence of comorbidities	2.83	0.022	1.16	6.92
Paternal educational attainment				
<High school	REF			
High school	0.66	0.638	0.12	3.66
Some college credits	0.52	0.497	0.08	3.45
College degree	0.96	0.961	0.21	4.49
≥Graduate degree	1.34	0.751	0.22	8.39
Annual household income				
Below sufficiency line	REF			
From sufficiency line to 100% above	1.29	0.612	0.48	3.51
>100% above sufficiency line	0.68	0.523	0.21	2.24
Parental Concerns Questionnaire (PCQ) Items				
My child does not use words or finds it difficult to initiate conversations with others.	1.16	0.633	0.64	2.09
My child always completes routines in the same way.	0.77	0.419	0.41	1.45
My child gets anxious in new or crowded places.	1.26	0.494	0.65	2.45
My child is sensitive to lights, sounds, and touch.	1.22	0.573	0.61	2.41
My child does not sleep easily or their sleep is interrupted.	1.39	0.226	0.81	2.38
My child hits or bites others.	1.11	0.686	0.66	1.86
My child is constantly moving, running, or jumping.	1.12	0.713	0.61	2.06
My child has difficulty completing the tasks assigned to them.	0.94	0.835	0.51	1.73
My child has sudden changed moods/emotions.	1.32	0.407	0.69	2.52
My child only eats certain types of food.	0.58	0.082	0.31	1.07
My child prefers to be alone or have few friends.	1.47	0.288	0.72	2.97
My toddler is shaking or flapping their hands.	0.79	0.386	0.46	1.35

## Data Availability

All the data for this study will be made available upon reasonable request.
